# Effectiveness of N95 Mask Fit Testing for the Prevention of Severe Acute Respiratory Syndrome Coronavirus 2: A Retrospective Case-Control Study

**DOI:** 10.7759/cureus.77168

**Published:** 2025-01-08

**Authors:** Goshi Fujimoto, Satomi Obikane, Kana Kuboyama

**Affiliations:** 1 Department of Gastroenterological Surgery, Koga Community Hospital, Yaizu, JPN; 2 Department of Infection Control, Koga Community Hospital, Yaizu, JPN

**Keywords:** covid-19, fit test, n95 mask, n95 respirator, sars-cov-2

## Abstract

Background

N95 masks can effectively prevent aerosol-transmitted infections. This study evaluated the ability of N95 mask fit testing to prevent severe acute respiratory syndrome coronavirus 2 (SARS-CoV-2), and explored risk factors associated with SARS-CoV-2.

Methods

Our hospital experienced two coronavirus disease 2019 (COVID-19) outbreaks. We conducted an N95 mask fit test that included 299 employees who were required to wear N95 masks for infection control. We analyzed the correlation between SARS-CoV-2 during the first cluster, and factors such as the fit test results. We also assessed the infection rates during the second cluster, based on the fit test results.

Results

Of the 265 employees who were included in the initial fit test, 154 (58.1%) passed. During the first cluster, the cumulative infection rate was 19.2% (51/265 employees). A significantly higher infection rate was observed among those who failed the initial fit test (p = 0.0264), and those with household COVID-19 cases (p = 0.00615). During the second cluster, a household COVID-19 case was the only significant risk factor (p = 0.0147) for infection.

Conclusions

Higher infection rates were observed among those who failed the N95 mask fit test, thus emphasizing the importance of proper mask use. N95 mask fit testing may help prevent SARS-CoV-2 infection.

## Introduction

The coronavirus disease 2019 (COVID-19) outbreak in 2019 occurred worldwide. In Japan, confirmed infections had to be reported to the nearest public health center, and measures of hospitalization and work restrictions were enforced by prefectural governors from February 1, 2020, to May 7, 2023. During the same period, seven peaks of infection were observed, with clusters also being reported at several medical facilities. Prevention of infection became an urgent priority, owing to the possibility of a severe shortage of medical staff as a result of infection among healthcare workers themselves.

In addition to vaccination, infection control measures such as refraining from going outside, wearing masks, promoting ventilation, and disinfecting hands and the surrounding environment were enforced. Healthcare workers followed the infection control measures imposed by their respective medical institutions and wore N95 masks when necessary. N95 masks can effectively prevent aerosol transmission of infections, and their use during surgery for patients with COVID-19 can reduce the risk of infection [1,2]. N95 masks are preferred over surgical masks for aerosol-generating procedures, or when a patient's COVID-19 status is positive or unknown [3]. Further, the U.S. and European Centers for Disease Control and Prevention (CDC) recommend the use of N95 masks, even for procedures that do not produce aerosols [4].

The disadvantages of N95 masks include the development of respiratory alkalosis and fatigue following mask use [5,6]. Therefore, it is imperative to ascertain the suitable subjects and conditions for the utilization of N95 masks. Proper usage of N95 masks requires appropriate knowledge and skills, with particular emphasis placed on the fit of the mask for the individual wearer. However, proper usage requires knowledge and skills, and a mask that fits the individual is essential. Fit testing evaluates the correct usage of masks; however, higher leakage rates have been observed among female and Asian healthcare workers [7]. Although healthcare workers are encouraged to fit test N95 masks to reduce the risk of infectious aerosols, few studies have evaluated whether fit testing reduces severe acute respiratory syndrome coronavirus 2 (SARS-CoV-2) infection. A retrospective study found no significant association between the use of fit-tested N95 masks and COVID-19 [8]. However, a direct comparison of the risk of SARS-CoV-2 infection with N95 mask fit testing has not been reported. During this study, we conducted N95 mask fit testing at our hospital and evaluated the ability of those masks to prevent SARS-CoV-2 infection. Our hospital experienced multiple COVID-19 clusters; therefore, we directly compared the risk of SARS-CoV-2 infection according to whether the fit test was performed or not performed. We also retrospectively examined risk factors for SARS-CoV-2 infection before and after fit testing during the cluster periods.

## Materials and methods

This retrospective case-control study was conducted over the course of 13 months. Our hospital experienced two COVID-19 clusters. Cluster 1 occurred between December 12, 2022, and February 22, 2023, and cluster 2 occurred between December 29, 2023, and January 29, 2024. Between June 21 and 29, 2023, a quantitative N95 mask fit test, which included 694 staff members, was performed. The study targeted 299 employees who were required to wear N95 masks as part of infection control measures between December 12, 2022, and January 29, 2024. This study was approved by the Ethics Committee of Koga Community Hospital, Yaizu, Japan (approval no.: 2024-7), and registered in the University Hospital Medical Information Network Clinical Trial Registry (UMIN000055914). The requirement for informed consent was waived because of the retrospective nature of the study. Anonymized data were used for this study.

A quantitative fit tester (MT-05U; SHIBATA, Tokyo, Japan) was used, and a leakage rate ≤10% was considered sufficient for passing the test. During the five-minute comfort assessment period, the mask position and mask strap adjustments were performed. Employees moved their heads up, down, left, and right, breathed, spoke, and performed a leak test. If they failed the leak test, then the fitting condition was rechecked, readjustments were performed, and the test was repeated. The fit test was administered by a certified infection control nurse. Nurses are eligible to complete the Japanese Nursing Association examination for infection control nurse certification after at least five years of obtaining a nursing license, at least three years working in the field of infection control, and the completion of at least 600 hours of certified nursing education, as defined by the Japanese Nursing Association. We examined the association between SARS-CoV-2 infection, which was defined as a positive nasopharyngeal swab antigen test result for SARS-CoV-2 performed as part of testing for symptomatic individuals or screening for asymptomatic individuals, during the cluster period, and various factors such as the fit test results. In addition, this study included 34 new employees who were not tested during the first cluster because they joined the staff after the testing period, thus allowing us to analyze infection rates based on the fit test results observed during the second cluster.

Statistical analysis

Data regarding SARS-CoV-2 infection, occupation, age, sex, household COVID-19 cases, and hand sanitizer use were retrospectively collected from reports provided to the hospital’s infection control office. SARS-CoV-2 infection was diagnosed using an antigen test. We explored the risk factors for infection during both cluster periods. Categorical variables were analyzed using Fisher’s exact test or the χ² test, as appropriate. Univariate and multivariate logistic regression analyses were performed to evaluate risk factors for SARS-CoV-2 infection, and statistical significance was considered when p < 0.05. All analyses were performed using EZR software (version 3.5.1) [9].

## Results

Of the 299 participants, 100 were male and 199 were female. Their occupations were as follows: emergency physician (n = 2); nurse, nursing assistant, or emergency medical technician (n = 207); rehabilitation staff member (n = 80); and clerk (n = 9). Of the 265 participants who underwent the initial fit test, 154 (58.1%) passed, and 111 (41.9%) failed. Twenty-two staff members (8.3%) were given different masks after failing three consecutive tests.

Exploration of SARS-CoV-2 infection risk factors during the first cluster

During the first cluster, 51 (19.2%) of 265 participants were infected. A univariate analysis revealed a significantly higher infection rate among those who failed the initial fit test (p = 0.0264) and those with household COVID-19 cases (p = 0.00615). No significant differences in sanitizer use (p = 0.11), age (p = 0.506), and sex (p = 0.629) were observed (Table 1).

**Table 1:** SARS-CoV-2 infection risk factors during the first cluster *A p-value of <0.05 is considered statistically significant. ^a^Data are presented as the median (range); ^b^Data are presented as the median (interquartile range). SARS-CoV-2, severe acute respiratory syndrome coronavirus 2

	Positive SARS-CoV-2 (n = 51)	Negative SARS-CoV-2 (n = 214)	T-value	p-value
Age, year^a^	36.5 (24-63)	34 (19-69)	-0.666	0.506
Sex, n (%)				0.629
Male	19 (36.5%)	81 (32.8%)	-	
Female	33 (63.5%)	166 (67.2%)		
Disinfectant, mL^b^	1400 (1040-1800)	1240 (800-1680)	1.607	0.11
Family members with infections				0.00615*
Positive	11 (21.2%)	13 (6.7%)	-	
Negative	41 (78.8%)	182 (93.3%)		
Fit test				0.0264*
Pass	37 (72.5%)	117 (54.7%)	-	
Fail	14 (27.5%)	97 (45.3%)		

Exploration of SARS-CoV-2 infection risk factors during the second cluster

During the second cluster, 31 (10.4%) of 299 participants were infected. A univariate analysis revealed a trend of lower infection rates for the group who underwent fit testing (p = 0.141). Infections were significantly higher among those with household COVID-19 cases (p = 0.0293) and women (p = 0.0426); no significant differences in age (p = 0.124) and sanitizer use (p = 0.717) were observed. A multivariate analysis indicated that a household COVID-19 case was the only significant risk factor for infection (p = 0.0147) (Table 2).

**Table 2:** SARS-CoV-2 infection risk factors during the second cluster *A p-value of <0.05 is considered statistically significant. ^a^Data are presented as the median (range); ^b^Data are presented as the median (interquartile range). CI, confidence interval; OR, odds ratio; SARS-CoV-2, severe acute respiratory syndrome coronavirus 2

	Positive SARS-CoV-2 (n = 31)	Negative SARS-CoV-2 (n = 268)	Univariate	Multivariate	
			p-value	OR (95% CI)	p-value
Age, year^a^	31 (24-60)	35 (19-69)	0.124	-	
Sex, n (%)			0.0426*		
Male	5 (16.1%)	95 (35.4%)	-		
Female	26 (83.9%)	173 (64.6%)	-		
Disinfectant (mL)^b^	1200 (840-4840)	1280 (850-1680)	0.717	-	
Family members with infections, n (%)			0.0293*	-	0.0147*
Positive	2 (6.5%)	1 (0.37%)	-	20.8 (1.81-238)	-
Negative	29 (93.5%)	267 (99.6%)	-	Reference	-
Fit test, n (%)			0.141	-	0.109
Performed	25 (80.6%)	240 (89.6%)	-	0.449 (0.169-1.20)	-
Not performed	6 (19.4%)	28 (10.4%)	-	Reference	-

## Discussion

SARS-CoV-2 is primarily transmitted via respiratory droplets. However, the virus has been found in aerosols and exhaled air; therefore, the potential for transmission through aerosols in certain situations exists [10]. N95 masks can effectively prevent aerosol-based transmission [1], as demonstrated by a study of 164 surgical staff members who wore N95 masks during 35 tracheostomies for patients with COVID-19 and reported no infections [2]. Studies have shown that the effectiveness of N95 respirators for preventing COVID-19 infection varies across countries. A study in Canada found that the hazard ratio for COVID-19 infection with the use of medical masks instead of N95 respirators was 2.83, which was the highest value among the countries studied [11]. Meanwhile, a study on 1009 healthcare workers in four countries providing direct care to COVID-19 patients reported that medical or surgical masks were as effective as N95 masks in preventing infection [12]. For influenza-like illnesses, including non-COVID-19 illnesses, N95/P2 masks may be more effective than surgical masks in preventing infection (relative risk (RR), 0.82; 95% confidence interval (CI), 0.66 to 1.03) [12]. Meanwhile, a meta-analysis of clinical studies up to 2014 found no statistically significant difference in the number of laboratory-confirmed respiratory infections between those using N95 masks and surgical masks [13]. The filtration efficiency of NaCl aerosol particles was 97.4% (±0.3%), whereas that of surgical masks was 18.4% [14]. During a virus aerosol exposure experiment using bacteriophage PhiX174, N95 masks that passed the fit test were associated with fewer particles found on the face (p = 0.007) and in nasal swabs (p = 0.058) compared to those associated with N95 masks or surgical masks that did not pass the fit test [15]. During studies of respiratory viruses and influenza, the infection rate of the group that used N95 masks was lower than that of the group that used medical masks (respiratory viruses: 1.4% vs. 2.6%; influenza: 0.3% vs. 1%). However, fit testing had no significant effect on the infection rate of the N95 mask group [16]. Although a retrospective study found no significant association between fit testing and COVID-19, “successful fit testing/checking” was performed for the masks used during the COVID-19 outbreak, which were the same masks as those used during fit testing after the outbreak; therefore, the fit test performance itself was not directly compared [8]. There are ethical issues associated with prospective comparisons of the impact of fit testing on SARS-CoV-2 infection. During our study, we were able to conduct a comparative study because new employees who had not performed fit testing began their employment during a specific period when clusters occurred multiple times.

During our study, participants who failed the fit test during the first cluster were not likely to wear their masks correctly and had significantly higher infection rates. The proper usage of N95 masks is essential for preventing infection. Although the group who underwent fit testing tended to have fewer infections during the second cluster, the difference was not statistically significant; this finding was possibly attributable to the time that had passed since the fit test (six months), thus leading to improper mask usage. However, after the initial fit test, the probability of fit test failure within three years was 0.6%; therefore, the six-month interval may have had little effect on the results [17]. Because we used a rented fit tester and did not perform a repeat fit test, the repeat confirmation of the proper use of N95 masks was not performed.

N95 mask availability was limited during the COVID-19 pandemic. Our hospital used masks distributed by the national and local governments. The staff members had no opportunity to select specific masks. Of the 265 participants who underwent fit testing, 22 (8.3%) failed three consecutive tests and were given different masks. Some participants failed because they were unfamiliar with adjustable masks. Therefore, an understanding of different types of N95 masks and their correct usage is essential. Previous studies have reported that the initial fit test pass rate ranged from 40% to 90%, consistent with the pass rate of 58.1% during our study [7]. Because of the high educational value of fit testing, it should be implemented as part of a hospital respiratory protection program [7]. At our hospital, fit testing is performed by infection control nurses who have received appropriate training.

Fit testing may be more important in areas where the weight of people changes frequently owing to their lifestyle, as changes in weight and face contour can result in fit test failures [7]. However, during the COVID-19 pandemic, comfort, price, and availability - rather than fit characteristics - often guided mask model selection, and the requirement for additional testing with each model change might have been cost-prohibitive in some regions [7]. In a study evaluating the cost-effectiveness of surgical and N95 masks in the prevention of COVID-19 among healthcare workers in India, the use of surgical masks yielded an annual cost saving of $1,390,713 per 10,000 healthcare workers compared to no mask use. Meanwhile, the use of N95 masks resulted in an annual cost saving of $63,919 per 10,000 healthcare workers compared to the use of surgical masks [4]. While N95 fit testing undoubtedly incurs additional costs, it has the potential to reduce overall healthcare costs by not only protecting healthcare providers, but also by taking into account the potential costs of sick leave and the legal costs associated with compensation [7]. Therefore, wearing fit-tested N95 masks is recommended from a cost perspective.

Participants with household COVID-19 cases had significantly higher infection rates during both clusters. Previous studies reported a secondary household infection rate of 23.0% [12]. Insufficient respiratory infection control and prolonged contact with infected household members may have accounted for this rate. Therefore, staff members with household COVID-19 cases should refrain from working to prevent the spread of infection.

This study also assessed the effectiveness of measures used to prevent infections attributable to contact, including frequent hand washing, disinfection with alcohol, and the use of gloves. Hand hygiene may be beneficial, with a relative reduction in respiratory illness of 11% (RR, 0.89; 95% CI, 0.83 to 0.94), reducing events from 200 per 1,000 to 178 per 1,000 (95% CI, 166 to 188) [12]. A previous study found that using chlorine or ethanol-based disinfectants at home had an effectiveness rate of 77% for preventing secondary household infections (odds ratio (OR), 0.23; 95% CI, 0.07 to 0.84) [18]. Although we quantitatively analyzed hand sanitizer use during this study, no significant differences were found, potentially because of the limited sample size.

This was a single-center study with a small sample size. Therefore, further studies with larger sample sizes are required. Additionally, the fit test was conducted only once during the cluster periods, and it was not possible to verify whether mask-wearing techniques were acquired through the fit test. Repeating the fit test may help healthcare workers acquire necessary mask-wearing techniques, resulting in fewer SARS-CoV-2 infections. This study examined the impact of fit testing involving a specific quantitative fit tester for SARS-CoV-2 infection during the COVID-19 outbreak; therefore, these results cannot be applied to diseases other than SARS-CoV-2 or to fit testing with other testers.

## Conclusions

N95 mask fit testing can reduce SARS-CoV-2 infections. However, further research is required to confirm the validity of this hypothesis. Selecting the correct mask and ensuring its correct usage are critical. The presence of household COVID-19 cases was a significant risk factor for disease transmission. Therefore, staff members with household COVID-19 cases should perform preventive measures to avoid disease transmission.
